# Fatty-Acid Composition and Sensory Traits of Japanese Local Chickens in the Context of Asian Local Chicken Meat Quality

**DOI:** 10.3390/ani16162588

**Published:** 2026-08-19

**Authors:** Hideaki Takahashi

**Affiliations:** National Agriculture and Food Research Organization (NARO), Tsukuba 305-8517, Ibaraki, Japan; takahashi8675@gmail.com

**Keywords:** Asian local chickens, JAS-certified chickens, poultry meat quality, rearing systems, native-breed ancestry, fatty-acid composition, arachidonic acid, sensory evaluation

## Abstract

Asian local chickens are often perceived as more palatable than commercial broilers, with stronger taste and longer aftertaste, but the biochemical basis of this eating quality remains unclear. Using Japanese local chicken meat as the main example and other Asian local chicken studies as context, this hypothesis-generating review develops a hypothesis centered on arachidonic acid. The core hypothesis is that higher arachidonic acid content may partly explain why local chicken meat is perceived as more palatable. Available Japanese data show that local chicken meats generally contain higher arachidonic acid proportions than commercial broiler meat. However, higher arachidonic acid content alone cannot explain why the meat is perceived as more palatable. A previously proposed hypothesis links lipid-derived volatile aldehydes to kokumi through activation of the calcium-sensing receptor. This route is treated cautiously here, because volatile aldehydes may be lost during cooking and have not yet been shown to remain in cooked chicken meat at concentrations that influence eating quality. Instead, this review proposes a second validation route. Arachidonic-acid-rich lipid pools generate 4-hydroxy-2-nonenal and related compounds that are less volatile and more likely to remain in edible fractions than short-chain aldehydes. These compounds are introduced here only as plausible downstream oxidation products that may help connect meat lipid composition with kokumi-related or aftertaste-related perception. This review emphasizes production context, meat-quality traits, and sensory evaluation as the primary basis for interpreting fatty-acid differences, with chemical hypotheses presented only as exploratory routes for future validation.

## Simple Summary

Local chicken meat is valued for its firm texture and distinctive taste, but these qualities do not arise by chance. They are influenced by chicken type, how long the birds are raised, how they are kept, what they are fed, and which part of the meat is evaluated. This review focuses on Asian local chickens, with particular attention to Japan, where naturally grown local chicken production is regulated by requirements for native-breed ancestry, rearing period, and husbandry conditions. The review explains how production factors can affect meat texture, fat composition, flavor-related traits, and sensory evaluation. It also emphasizes that possible chemical explanations for flavor should be tested only after clear differences in meat quality and sensory properties have been shown.

## 1. Introduction

Asian local chicken meat offers a useful model for examining why meat from some chicken production systems is perceived as more palatable than commercial broiler meat. Its market value is commonly associated with stronger taste, longer aftertaste, firm texture, lipid composition, and regional product identity [1]. These attributes should not be interpreted from product image alone. They need to be connected to chicken type, production conditions, meat composition, cooking-derived chemistry, and sensory evidence.

Indigenous and local chicken systems remain important throughout South and East Asia. Studies from Thailand [2,3,4], China [5,6,7], and Republic of Korea [8,9] show that these birds can differ from commercial broilers in carcass traits, muscle development, physicochemical meat quality, fatty-acid composition, volatile profiles, metabolite patterns, and sensory-related traits. These findings indicate that local chicken meat quality is not merely a matter of consumer preference or regional image, but a biologically grounded phenotype shaped by chicken type, genetic resources, and management systems.

Japanese local chicken meat provides the main example in this review because part of this production sector is defined by the Japanese Agricultural Standard (JAS). The JAS-certified chicken standard requires at least 50% native-breed ancestry, a rearing period of at least 75 days, and floor-rearing from 28 days of age under defined stocking-density conditions [10]. These criteria do not explain flavor by themselves, but they provide a regulated production context in which meat-quality traits can be compared with those of commercial broilers. Brands such as Hinai-jidori, Tokyo Shamo, Nagoya Cochin, Hakata Jidori, Aizu-jidori, Amakusa Daioh cross, and Miyazaki Jitokko are treated here as examples within this Japanese local chicken framework.

Arachidonic acid (AA) is a useful biochemical clue in this context, but it cannot by itself explain local chicken palatability. Comparative studies and compiled Japanese local chicken datasets report differences between Japanese local chickens and commercial broilers in fatty-acid composition, including AA and linoleic acid (LA) levels [11,12,13,14,15]. Feeding studies have also shown that increasing AA content in chicken thigh meat can improve sensory attributes such as taste intensity, continuity, aftertaste, and overall palatability [16,17]. The key unresolved issue is how, and under what experimental conditions, this fatty-acid difference should be connected to measurable meat-quality traits, sensory responses, lipid-derived compounds, and possible receptor-oriented mechanisms.

Existing studies often address these elements separately: production studies describe chicken type, dietary lipid source, growth, or meat composition; sensory studies evaluate palatability; and flavor-chemistry studies measure volatile or oxidation-derived compounds. As a result, the available studies still do not show how production traits, AA status, sensory differences, and later chemical or receptor-based tests should be connected in the same experimental logic. This review therefore keeps that order explicit: rearing conditions and meat-composition traits are defined first, sensory differences are examined next, and lipid-derived compounds or receptor-oriented hypotheses are considered only after those earlier links have been established. This ordering avoids treating AA, volatile compounds, or receptor activation as independent explanations of palatability.

Cooking models are useful in this context only when they standardize sample preparation for meat-quality and sensory evaluation. A simmered broth system is considered here as one controlled model for testing whether production-derived differences in meat composition, texture, and sensory traits remain detectable in an edible matrix, rather than as a subject of culinary practice or regional food culture.

Accordingly, this review uses Japanese local chicken meat as the main example to ask whether higher AA content can help explain the perceived palatability of Asian local chickens. AA is presented as a candidate biochemical clue rather than a complete flavor explanation. The review first places this clue within the broader production context of chicken type, rearing system, growth, dietary lipid source, meat portion, texture, and sensory evaluation. Lipid-derived volatile compounds, cooking-derived oxidation products, and receptor-oriented hypotheses are then considered only after production-related meat-quality and sensory differences have been established. This review proposes that higher AA content may contribute to the perceived palatability of local chicken meat, not as an isolated fatty acid but through its metabolic balance with linoleic acid. Because AA and LA compete within n-6 oxidation pathways, the AA/LA ratio may influence the relative formation of volatile aldehydes and nonvolatile lipid-oxidation products, thereby shaping kokumi-related and aftertaste-related sensory responses.

## 2. Review Strategy and Scope of Literature Use

This article is a hypothesis-generating narrative review, not a systematic review or meta-analysis. The literature was selected to clarify how production conditions in Japanese local chicken systems relate to measurable meat-quality traits, fatty-acid composition, and sensory outcomes, with additional comparative material from other Asian local chicken studies where relevant. The literature was identified primarily through searches of PubMed, Web of Science, Google Scholar, and J-STAGE. Searches focused on studies published between approximately 2000 and 2026 using terms related to local chickens, production systems, fatty-acid composition, meat quality, lipid oxidation, and sensory evaluation. Because this review is narrative, studies were chosen based on conceptual relevance to production context, meat-quality traits, and sensory outcomes rather than on systematic inclusion criteria. Emphasis was placed on studies dealing with chicken type, native-breed ancestry, regulated production categories, rearing period, housing system, dietary lipid source, growth, muscle development, carcass traits, physicochemical meat quality, fatty-acid composition, lipid oxidation, and sensory evaluation. JAS-certified chickens are used as the main Japanese reference point, with other Asian indigenous and local chickens providing supplementary comparative context.

Studies on cooking-derived lipid oxidation, broth matrices, volatile aldehydes, nonvolatile lipid-oxidation products, and receptor-oriented mechanisms were considered more selectively. They were included only when they helped explain how meat-composition or sensory differences might later be examined chemically or mechanistically. This review is not meant to cover poultry flavor chemistry, cooking practice, or receptor biology in full. Rather, it brings those areas in only where they help connect chicken type, production conditions, dietary lipid source, fatty-acid composition, and instrumental meat-quality traits with sensory evaluation and, when supported by the evidence, chemical or receptor-oriented follow-up.

## 3. JAS-Certified Chickens as a Regulated Production Framework for Meat-Quality Evaluation

The JAS category gives local chicken meat a defined production basis for meat-quality evaluation. Under the JAS standard, birds must have at least 50% native-breed ancestry, be reared for at least 75 days after hatching, and be floor-reared from 28 days of age at a stocking density of 10 birds or fewer per square meter [10]. These requirements define Jidori meat by ancestry, rearing period, and husbandry conditions. For this reason, JAS-certified chicken meat should be examined first as a defined production category, not simply as a regional food image or culinary label. These criteria are also useful when interpreted in relation to Japanese native chicken genetic resources and earlier hypothesis-based discussions of poultry meat palatability [11,12].

Genotype, slaughter age, diet, and rearing system are treated as distinct production variables in this review, because each influences meat quality through different biological pathways. Their effects cannot be interpreted interchangeably and must be evaluated under matched analytical and sensory conditions.

Genetic factors (native-breed ancestry, growth pattern, and desaturase activity) are treated separately from management factors (rearing period, stocking density, activity, and diet) in this review, because they influence meat quality through different biological mechanisms. Their contributions are therefore interpreted as distinct and non-interchangeable components of the production system.

Although JAS categories define production conditions and marketing standards, they do not themselves specify biological differences. Therefore, any physiological, fatty-acid, or sensory differences discussed in this review are interpreted as biological outcomes of production practices rather than regulatory definitions.

Each certification requirement also corresponds to variables with plausible effects on carcass and meat traits. Native-breed contribution may be associated with differences in growth pattern, muscle development, lipid deposition, and meat composition. A longer rearing period may affect body weight, muscle maturation, collagen characteristics, texture, and postmortem meat quality. Floor-rearing and stocking density may alter activity, management context, muscle use, and carcass traits. Certification alone does not demonstrate a flavor mechanism; instead, it defines the production context within which meat-quality traits, including fatty-acid composition, should be interpreted.

Within the JAS framework, the rearing period of at least 75 days should be viewed as an important production variable defined by the standard [10], but not as direct evidence of a specific biological effect. Its possible relationships with muscle maturation, texture, fatty-acid composition, and sensory traits are evaluated in later sections together with genotype, diet, rearing system, meat portion, and analytical conditions [18,19,20,21,22,23,24].

Within this context, fatty-acid composition should be regarded as one measurable meat-quality trait, not as a self-sufficient explanation of palatability. Comparative studies and controlled dietary studies indicate that arachidonic acid and linoleic acid levels can differ between local chickens and commercial broilers and can be modified experimentally through diet [11,12,13,14,15,16,17]. Fatty-acid profiling can therefore help bridge production context, meat composition, and sensory evaluation. Its interpretation, however, should remain integrated with meat portion, texture, postmortem handling, and sensory-panel outcomes.

The role of JAS-certified chickens in this review is to provide defined production examples for testing meat-quality variables. Because individual brands differ in genetic and production background, region, diet formulation, rearing duration, and product specification, these factors should be treated as sources of experimental variation. Interpretation should therefore proceed from production traits to carcass and meat-quality traits, and then to fatty-acid composition, texture, and sensory evaluation.

## 4. Fatty-Acid Composition as a Meat-Quality Trait in JAS-Certified Chickens

Building on the JAS production context introduced above, this section uses the previously reported Hinai-jidori–broiler comparison only as an entry point for examining fatty-acid composition across selected JAS-certified chickens [11,14,15]. Table 1 summarizes patterns in AA, LA, and AA/LA across selected JAS-certified chicken entries rather than describing individual brands.

The Table 1 pattern is supported by additional examples. Aizu-jidori breast meat has been reported to contain a higher AA proportion than broiler breast meat [12], and Choshu-Kurokashiwa thigh meat showed AA enrichment together with higher sensory scores for meaty odor, meaty flavor, umami taste, and aftertaste intensity [13]. Together, these data indicate that the profile shifted toward higher AA is not limited to Hinai-jidori and is worth testing across JAS-certified chickens under matched analytical conditions.

Comparable findings from Thailand [3,4], China [5,6,7], and Korea [8,9] further show that chicken type and production context are associated with differences in carcass traits, meat quality, fatty-acid composition, metabolites, volatile compounds, and sensory-related characteristics. Within this animal-science framing, fatty-acid composition is best treated as a measurable meat-quality trait within a production context, to be interpreted with growth, rearing period, dietary lipid source, meat portion, intramuscular fat, texture, lipid-oxidation indices, and sensory-panel outcomes.

AA/LA is therefore used in this review only as a directional interpretive variable, not as a predictive index of flavor quality. Its relevance depends on matched cooking conditions, controlled volatile-capture protocols, and matrix-specific oxidation behavior. AA/LA indicates substrate availability for pathways generating compounds such as 2,4-decadienal, but the sensory impact of these volatiles must be confirmed empirically through targeted chemical analysis and sensory evaluation. For this reason, AA/LA is treated as a contextual comparison tool rather than a universal indicator of palatability.

AA levels observed in JAS-certified chickens arise from production-system factors such as genotype, rearing duration, diet, and physiological maturation, whereas AA-supplementation studies elevate AA through targeted dietary intervention under controlled experimental conditions. For this reason, supplementation studies are used in this review only as physiological reference evidence rather than as directly comparable outcomes. Their role is to illustrate the sensory and volatile consequences of experimentally increasing AA, not to imply equivalence with production-derived AA variation.

Takahashi et al. [17] provide an experimental reference point for interpreting the possible sensory relevance of AA variation in broiler thigh meat. In that feeding study, broilers received a corn-oil control diet or diets containing graded levels of AA-enriched oil for one week before slaughter, after which skinless thigh meat was analyzed for fatty-acid content and sensory attributes. Thigh-meat AA increased from 0.57 ± 0.05 mg/g in the corn-oil control group to 0.98 ± 0.05 mg/g in the 1/4 AA-enriched-oil group, an approximately 1.7-fold increase. When recalculated as a proportion of the quantified fatty acids reported in Table 2 of that study, AA increased from approximately 0.9% to 1.9%, or about 2.1-fold. In the corresponding pairwise sensory comparison of steamed minced thigh meat, the 1/4 AA-enriched-oil group scored higher than the corn-oil control group for flavor intensity, umami, kokumi, and aftertaste. Because the approximately two- to four-fold higher AA proportions in selected JAS-certified chicken entries in Table 1 are of equal or greater magnitude than this experimentally detectable AA range, they should not be dismissed as merely descriptive chemical variation. Even so, the feeding-study evidence comes from steamed minced broiler thigh meat. It supports sensory relevance by magnitude, but it does not directly prove the same effect in intact meat, breast meat, or all JAS-certified chicken portions.

**Table 2:** Candidate nonvolatile lipid-oxidation markers for downstream validation in cooked edible fractions.

Property	4HNE	4HHE
Main precursor origin	Arachidonic-acid-related omega-6 hydroxy fatty-acid autoxidation within n-6 lipid pools shifted toward arachidonic acid	n-3 polyunsaturated-fatty-acid oxidation, especially docosahexaenoic-acid- and eicosapentaenoic-acid-related lipid peroxidation
Relationship to meat lipid traits	Candidate downstream marker that may reflect an upstream n-6 lipid pool shifted toward arachidonic acid relative to linoleic acid	Candidate comparator for determining whether n-3-derived oxidation products should also be measured
Volatility under cooking	Low or nonvolatile under ordinary cooking conditions	Low or nonvolatile relative to short-chain aldehydes
Matrix behavior	Amphiphilic and electrophilic; may be retained at lipid–water interfaces, meat surfaces, dispersed fat droplets, and protein- or peptide-rich cooked fractions	Amphiphilic and electrophilic; similar retention is possible, with relevance depending on n-3 polyunsaturated-fatty-acid abundance
Protein/peptide adduction	Can form cysteine-, histidine-, and lysine-associated adducts that may retain electrophilic products in edible fractions	Potentially similar adduct-forming behavior; included mainly for comparative measurement
Main analytical role	Candidate nonvolatile n-6-derived marker for downstream chemical validation	Optional n-3-derived comparator for downstream chemical validation
Receptor-oriented interpretation	Optional future validation target only if direct edible-fraction measurements and sensory data justify testing	Optional comparator; not central unless n-3-derived oxidation is biologically or analytically relevant
Status in the present review	Candidate downstream marker requiring direct quantification in standardized cooked edible fractions	Secondary comparator, not central unless diet, chicken type, or production conditions alter n-3 polyunsaturated-fatty-acid-derived oxidation

Note: C3–C6 aldehydes are summarized in Table 3 as volatile lipid-oxidation markers. 4HNE and 4HHE are listed here only as candidate nonvolatile markers for future measurement, not as compounds already detected in cooked local chicken samples [25,26,27,28].

**Table 3:** Candidate short-chain aldehydes derived from poultry meat lipid oxidation and their expected usefulness as volatile markers in standardized aqueous cooking models.

Property	Propanal (C3)	Butanal (C4)	Pentanal (C5)	Hexanal (C6)
Main lipid-origin relevance	More strongly associated with n-3 PUFA oxidation	Less central to the n-6 PUFA hypothesis	Frequently detected among n-6 PUFA-derived aldehydes	Major LA-derived oxidation product
Boiling point	~48 °C	~75 °C	~102–103 °C	~128–131 °C
Water solubility	Highly water-soluble; approximately 200–300 g/L at 20–25 °C	Moderately soluble; approximately 70–80 g/L at 20–25 °C	Slightly soluble; approximately 14 g/L at 20–25 °C	Slightly soluble; approximately 5–6 g/L at 20–25 °C
Expected behavior in standardized aqueous cooking models	Water-accessible but rapidly volatilized in open near-boiling conditions; sustained aqueous accumulation is unlikely	Water-accessible but readily lost to the headspace; unlikely to persist at receptor-relevant aqueous concentrations	Intermediate volatility and measurable water compatibility; analytically monitorable, but open boiling is expected to prevent micromolar aqueous accumulation	Lower volatility than C3–C5 and continuously generated from LA-rich oxidation; useful marker, but aqueous persistence remains limited by lipid partitioning, headspace transfer, and secondary reactions

Note: Values are approximate database-derived physicochemical descriptors and should not be interpreted as experimentally measured solubilities under actual standardized chicken-cooking conditions. Boiling points and water-solubility descriptors, including approximate water-solubility values for C3–C6 aldehydes, were compiled from PubChem and the NIST Chemistry WebBook [29,30]. The table is intended to compare relative volatility, water compatibility, lipid-origin relevance, and expected phase behavior during aqueous cooking used for analytical evaluation.

## 5. Growth, Rearing Period, Genotype, and Meat Texture

Growth rate, slaughter age, and rearing conditions are major determinants of poultry meat quality, and local or slow-growing chickens often differ from fast-growing broilers in carcass traits, muscle structure, shear force, collagen characteristics, texture, water-holding capacity, cooking loss, fatty-acid composition, and sensory attributes [18,19,20,21,22,23,24].

In JAS-certified chickens, the rearing period of ≥75 days should therefore be considered a production variable that may contribute to muscle maturation and texture-related traits, although it should not be interpreted as a direct biological mechanism by itself [10]. Slow-growing or native-breed chickens accumulate muscle mass over a longer physiological timeline, and age- and genotype-associated differences can contribute to firmer texture, greater springiness, and altered postmortem meat-quality traits [18,19,20,21,22,23,24,31,32]. Genotype-related lipid metabolism may also affect intramuscular lipid deposition, membrane lipid remodeling, and fatty-acid composition [21,22]. These lipid traits should be evaluated alongside rearing-period-related texture traits and sensory outcomes before perceived palatability is attributed to lipid-derived compounds alone.

Genotype-related differences in desaturase activity also support this interpretation. Slow-growing chickens exhibit higher hepatic FADS1 and FADS2 expression and greater Δ5/Δ6 desaturase activity than fast-growing birds, resulting in higher PUFA content, including n-6 derivatives such as arachidonic acid in breast meat [21].

These production-related texture and lipid-metabolism traits provide the biological context for considering downstream lipid-oxidation products. In this review, fatty-acid differences are therefore not treated as independent flavor explanations, but as meat-composition traits that should be interpreted only after growth, rearing period, muscle development, texture, and sensory endpoints have been evaluated.

The AA → 4HNE oxidation pathway is therefore treated in this review as a supported biochemical mechanism [33,34]. However, its quantitative and sensory implications in cooked local chicken meat remain exploratory and require matched analytical validation before any production-related interpretation can be made.

The longer rearing period required for JAS-certified chickens [10], together with reports of firmer texture and stronger sensory attributes related to palatability in local chicken meat [1,2,3,4,5,6,7,8,9], makes cooking conditions important for sensory interpretation. Dry heating may accentuate toughness when sample preparation is not controlled, although the response also depends on postmortem myofibrillar protein changes and processing conditions [23,24]. Aqueous simmering offers a useful alternative experimental model. It allows meat-derived compounds to enter a broth-like matrix while keeping heating conditions sufficiently standardized for comparing firm-textured local chicken meat with broiler meat. A simmered chicken broth system is considered here only in that analytical sense [19].

Future comparisons should therefore include growth performance, slaughter age, muscle-fiber development, collagen characteristics, shear force, cooking loss, and postmortem protein changes in both local chickens and broilers. Descriptive sensory analysis should be paired with instrumental texture measurements, because descriptors such as hardness, springiness, chewiness, and mouthcoating can separate local or native chicken meat from broiler meat and are associated with shear force and collagen-related traits [24]. Because reported texture values are strongly affected by muscle portion, cooking endpoint, sampling direction, and shear-force protocol, future comparisons should report absolute instrumental values together with standardized methods rather than relying only on sensory descriptors. These measurements are needed to show whether perceived palatability is rooted in bird growth and muscle development, rather than being attributed only to cooking or broth composition.

## 6. Dietary Lipid Source, Lipid Metabolism, and Fatty-Acid Composition

Dietary lipid source is one experimental tool for testing whether meat lipid composition contributes to palatability. In chicken meat, intramuscular lipid content, lipid class distribution, and fatty-acid composition vary with chicken type, genetic line, growth rate, age, meat portion, and diet. For local chicken systems, these variables connect live-bird management with the chemistry of cooked meat. Among these variables, dietary lipid source is especially informative because it can alter fatty-acid profile, lipid indices, oxidative stability, and sensory-related traits in broiler meat [35,36]. AA has been discussed as a palatability-related fatty acid in chicken meat [16,17,23], whereas LA is a major precursor of hexanal and related oxidation products in lipid-oxidation and meat-flavor systems [37,38,39,40]. The AA/LA value is therefore used here not as a simple ratio for description, but as a lipid trait that may summarize differences associated with chicken type, rearing system, meat portion, and dietary lipid source [11,14,15,16,17,18,31,32,35].

Table 1 shows that AA/LA separates broiler breast meat from the selected JAS-certified chicken entries more clearly than AA proportion alone. Broiler breast meat had an AA/LA value of 0.24, whereas all listed JAS-certified entries were higher. Tokyo Shamo, Nagoya Cochin, Hinai-jidori, Hakata Jidori, and Aizu-jidori formed a relatively narrow high-AA/LA range of 0.59–0.68. Amakusa Daioh cross showed the largest shift, with a breast AA/LA value of 0.89, reflecting high AA against a moderate LA background. Miyazaki Jitokko was lower than the other listed JAS-certified chickens, but still exceeded broiler breast meat at 0.40. Thus, the selected JAS-certified entries are not compositionally identical, yet each shows a breast lipid profile shifted toward AA relative to LA compared with broiler meat.

A similar pattern appears in thigh meat, but the separation is weaker. Broiler thigh meat had an AA/LA value of 0.11, and all selected JAS-certified entries were higher. Tokyo Shamo showed the highest thigh value at 0.28, followed by Hakata Jidori, Amakusa Daioh cross, and Aizu-jidori. Hinai-jidori was intermediate, whereas Nagoya Cochin and Miyazaki Jitokko were lower, although still above the broiler value. In every chicken type, however, thigh AA/LA was lower than the corresponding breast value. The profile shifted toward higher AA is therefore present in both portions, but is more pronounced in breast meat and partly attenuated in thigh meat by the larger LA pool.

## 7. Volatile Lipid-Oxidation Markers as Downstream Traits in Production-Related Meat-Quality Assessment

Lipid-derived aldehydes, alcohols, ketones, and related volatiles arise from lipid composition, storage, heating, and oxidation, and are widely used in aroma-related evaluation of meat quality [37,38,39]. Oxidation of unsaturated fatty acids produces hydroperoxides that subsequently decompose into secondary volatile products, including aldehydes, alcohols, and ketones [40,41,42,43,44]. In the present discussion, C3–C6 aldehydes are used mainly as measurable signs of lipid oxidation and aroma formation when local chicken and broiler meat are compared under defined analytical conditions. The chemical content in this section is included only to outline downstream oxidation markers that may later be tested under controlled conditions. These descriptions are not intended as full mechanistic treatments but as exploratory links connecting production-related lipid traits with measurable analytical outcomes.

### 7.1. Lipid Origin of C3–C6 Aldehydes

In poultry meat-quality research, C3–C6 aldehydes provide measurable oxidation volatiles produced from meat fatty acids during storage and cooking. Kitajima et al. [38] showed that defined C3–C6 aldehydes can activate the calcium-sensing receptor under controlled in vitro and sensory-test conditions. In a production-centered setting, these compounds are most useful for comparing lipid stability, aroma-related traits, and cooking-derived volatile profiles among chicken types, portions, diets, and rearing systems.

Most published sensory evidence related to arachidonic-acid enrichment has been obtained from thigh meat. Therefore, these findings are referenced here only as supportive context and should not be directly generalized to breast-meat AA/LA patterns without cut-specific validation.

Short-chain aldehydes arise mainly through oxidative degradation of polyunsaturated fatty acids. In chicken meat, arachidonic acid and linoleic acid are relevant because n-6 polyunsaturated-fatty-acid oxidation can yield pentanal, hexanal, and related compounds, whereas propanal is more often associated with n-3 polyunsaturated-fatty-acid oxidation [37,38,39,40]. Heating temperature and fatty-acid type can further alter the formation of lipid-oxidation products during thermal processing [41], and cold storage and heat treatment of chicken meat can influence oxidative stability and oxidation-related marker profiles [42,43]. Together, these compounds help describe aroma formation, oxidative stability, and production-related meat-quality differences.

Interpretation of lipid-derived aldehydes requires controlled storage and heating conditions. Because chicken meat is cooked under aerobic conditions in both simmering and dry-heat methods, oxygen exposure is treated here as a controlled boundary condition rather than as a production variable. Antioxidant status, including vitamin E content, also modulates lipid-oxidation kinetics and is therefore treated as an analytical boundary condition that must be controlled when evaluating production-related differences in aldehyde formation.

Hexanal is a well-established product of linoleic acid oxidation and is widely used as a marker of lipid oxidation in food and meat systems [37,38,39,40]. Pentanal is also commonly detected during oxidation of polyunsaturated-fatty-acid-rich lipids, and its measured abundance can vary with thermal processing and oxidative conditions [41,43]. For this review, C5/C6 aldehyde patterns are treated as downstream markers of lipid oxidation and aroma-related meat quality. A higher arachidonic-acid-to-linoleic-acid value in local chicken meat may alter volatile aldehyde profiles under defined heating and oxidative conditions, and this possibility can be tested by paired fatty-acid and volatile analyses.

In this review, volatile aldehydes such as pentanal and hexanal are treated as analytical oxidation markers, whereas compounds with potential receptor activity (e.g., 4HNE or CaSR-active C3–C6 aldehydes) are considered separately as hypothesis-generating sensory-active candidates. These categories are not interchangeable and are interpreted under different experimental assumptions.

The 4HNE–TRPA1 pathway is included here as a narrative, hypothesis-generating link between lipid oxidation and potential oral sensory responses. Although the biochemical basis of 4HNE formation is well established, its direct contribution to sensory perception in cooked poultry meat remains to be validated under controlled experimental conditions.

The pentanal-to-hexanal ratio can therefore be used as a working index of volatile oxidation balance, but not as a conversion ratio. In this review, the pentanal/hexanal ratio is treated strictly as an exploratory indicator of oxidation balance rather than as a mechanistic or predictive marker. Its interpretation requires paired fatty-acid data, standardized heating conditions, and controlled sampling, and therefore remains provisional until validated under matched analytical frameworks. AA/LA describes upstream precursor composition. By contrast, pentanal-to-hexanal reflects precursor availability together with oxidation pathway, heating condition, volatilization, degradation, and phase partitioning [29,30,37,39,40,41]. If local chicken meat repeatedly shows different pentanal-to-hexanal values or broader C5/C6 aldehyde patterns from broiler meat under matched conditions, volatile aldehydes would be useful production-related aroma and oxidation markers.

The linoleic acid literature is cited here mainly to support hexanal-centered volatile-marker interpretation. It should not be read as defining the nonvolatile oxidation-marker argument developed in Section 8.

Studies in native or local chicken systems provide indirect support for this interpretation. Work on the Taihe silky chicken [3], the Yanjin blackbone chicken and the Piao chicken [5], the Beijing Youji chicken [6], and the Korean native Woorimatdag No. 2 chicken [9] shows that indigenous or local chicken systems can differ from commercial broilers, or from one another, in polyunsaturated-fatty-acid composition, small-molecule metabolites, lipid-oxidation indices, and volatile aldehyde patterns. These reports support the broader view that chicken type and production context are associated with differences in lipid composition and oxidation-derived volatile profiles.

A gas chromatography–ion mobility spectrometry study of four Chinese local chicken types gives a related example of chicken-type-associated variation in volatiles [44]. Approximate pentanal-to-hexanal values calculated from the reported signal intensities differed among the chicken types examined. Because that study lacked broiler controls and paired fatty-acid data, the differences should be interpreted only as chicken-type-associated volatile variation. They cannot be assigned to arachidonic acid, linoleic acid, or AA/LA without matched lipid-composition measurements.

Future studies should measure fatty acids and volatile aldehydes in the same matched samples so that upstream lipid traits can be tested against downstream volatile-marker profiles.

### 7.2. Standardized Cooking Models for Evaluating Volatile Lipid-Oxidation Markers

Standardized cooking models are useful because they control sample preparation when meat from different production systems is compared. Aqueous heating is treated here as an analytical model, not as a culinary subject. The aim is to test whether differences in lipid composition, oxidative stability, meat portion, and fat content appear as measurable volatile profiles under matched heating and sampling conditions.

During aqueous heating, meat-derived lipids and volatile oxidation products partition among aqueous, lipid, interfacial, and headspace phases. Table 3 is therefore methodological: it compares lipid-origin relevance, volatility, water compatibility, and expected phase behavior of C3–C6 aldehydes during standardized aqueous cooking. Pentanal, hexanal, and related aldehydes are prioritized as practical markers of lipid oxidation, oxidative stability, and aroma-related meat quality [37,38,39,40]. Their measured concentrations can be affected by heating temperature, fatty-acid type, storage, heat treatment, and chicken-matrix conditions [41,43], as well as by formation, volatilization, degradation, lipid and headspace partitioning, secondary reactions, analytical recovery, and physicochemical properties [29,30]. These variables must be controlled before volatile differences are attributed to chicken type, dietary lipid source, rearing system, or meat portion.

In practice, C3–C6 aldehydes are most useful as volatile signs of lipid oxidation and aroma formation under controlled cooking and sampling conditions. They show whether differences in lipid composition, oxidative stability, storage, heating, and sample preparation appear as measurable volatile profiles. Although defined short-chain aldehydes can activate the calcium-sensing receptor under controlled assay conditions [38], extending this evidence to sensory or receptor-based interpretation requires direct edible-fraction measurements, relevant concentrations, and sensory-panel data.

## 8. Nonvolatile Lipid-Oxidation Products in Downstream Meat-Quality Validation

The preceding section considered C3–C6 aldehydes as volatile indicators of lipid oxidation and aroma-related meat quality. Nonvolatile lipid-oxidation products require a separate treatment because some electrophilic aldehydes may remain in cooked edible fractions rather than being lost to the headspace. In this section, 4-hydroxy-2-nonenal (4HNE) and 4-hydroxy-2-hexenal (4HHE) are discussed as compounds that need to be measured directly in cooked edible fractions, rather than as sensory-active compounds already demonstrated in chicken meat.

For the present argument, it is useful to separate the fatty-acid profile from the oxidation products that might arise during cooking. AA/LA describes the meat lipid profile within a production context, whereas 4HNE and 4HHE would need to be measured directly in standardized cooked edible fractions.

### 8.1. Chemical Characteristics of 4HNE and 4HHE as Candidate Markers

4HNE and 4HHE are hydroxyalkenals formed during lipid oxidation. Chemically, they differ from simple volatile aldehydes such as propanal, pentanal, or hexanal because they contain both a hydroxyl group and an α,β-unsaturated carbonyl structure. Their low volatility, amphiphilic character, and electrophilic reactivity make them plausible markers of nonvolatile oxidation in cooked edible fractions. The point is analytical rather than sensory-mechanistic.

This separation also keeps the chemical discussion tied to meat-quality testing. Volatile aldehydes are useful mainly for aroma-related oxidation, whereas 4HNE and 4HHE may indicate electrophilic products that remain in edible fractions. Their usefulness should be checked against chicken type, production conditions, dietary lipid source, meat portion, and fatty-acid composition.

### 8.2. Fatty-Acid Origins and Upstream Meat Lipid Traits

The fatty-acid origin of these compounds has to be read against the meat lipid profile. Mechanistic work shows that omega-6 hydroxy linoleic and arachidonic acids can be transformed autoxidatively into chiral 4-hydroxy-2E-nonenal [34]. Because Table 1 shows higher AA proportions and AA/LA values in selected JAS-certified chicken entries rather than in broiler meat, 4HNE is a reasonable compound to measure when testing whether n-6 lipid pools shifted toward arachidonic acid lead to retained oxidation products in cooked meat. This does not mean that 4HNE is assumed to be present or sensory-active in cooked local chicken meat. By contrast, 4HHE is more closely associated with n-3 polyunsaturated-fatty-acid oxidation, particularly docosahexaenoic acid and related n-3 hydroperoxy derivatives [25]. AA/LA is therefore used only as an upstream indicator of an n-6 lipid pool shifted toward arachidonic acid that requires direct downstream testing.

This argument still needs direct poultry-meat evidence. The poultry literature supports differences in fatty-acid composition between selected JAS-certified chickens and broilers, but it does not show that 4HNE or 4HHE reaches sensory-relevant concentrations after cooking. Future studies should measure lipid traits and nonvolatile oxidation products in the same samples. 4HHE should be included mainly as a comparator when diet, chicken type, or production conditions suggest meaningful n-3 PUFA variation.

### 8.3. Retention in Standardized Cooked Edible Fractions

The analytical value of 4HNE and 4HHE depends on retention as well as formation. Unlike short-chain volatile aldehydes, these low-volatility electrophiles may remain near lipid droplets, meat surfaces, protein-rich components, gelatinized collagen fragments, or lipid–water interfaces. This behavior makes them suitable candidates for measurement in cooked meat and cooking-loss fractions, provided that free and bound forms are distinguished under controlled conditions.

In aqueous cooked chicken samples, dispersed fat and lipid droplets can act as local sites for hydroperoxide decomposition and secondary aldehyde formation. Proteins, peptides, gelatin, collagen fragments, and amino-acid side chains may also trap 4HNE through reactions with cysteine, histidine, and lysine residues [26,27,28]. These reactions can lower free 4HNE concentrations while retaining 4HNE-related products within the edible matrix. Future studies will need to distinguish free, lipid-associated, and protein- or peptide-adducted forms instead of relying only on total concentration.

The main experimental question is whether differences in meat lipid composition remain visible as measurable nonvolatile oxidation products after standardized cooking.

Because the selected JAS-certified chicken entries in Table 1 show higher AA proportions and AA/LA values than broiler meat, standardized aqueous cooking provides a practical model for testing whether lipid pools shifted toward AA are associated with higher levels of 4HNE-related nonvolatile products under matched conditions. The thigh-meat feeding studies remain useful for judging magnitude, but they do not prove effects across meat portions.

### 8.4. Role in Future Meat-Quality Validation Studies

AA/LA is most useful as an upstream descriptor of the meat lipid profile in production-based meat-quality evaluation. It may show whether n-6 lipid composition differs among production categories, portions, dietary lipid sources, and rearing systems, but it does not demonstrate formation, retention, sensory relevance, or receptor activity of any downstream product. Links between AA/LA and nonvolatile oxidation products therefore require matched fatty-acid profiling, oxidative-stability measurements, and direct quantification of candidate markers in standardized cooked edible fractions.

Quantitative estimates may help design experiments, but they should not be treated as sensory exposure data. Concentrations of 4HNE, 4HHE, and related adducts will depend on precursor abundance, cooking and storage conditions, matrix binding, degradation, sampling, and analytical recovery. These measurements should be interpreted alongside growth performance, carcass traits, meat portion, fatty-acid composition, oxidative stability, instrumental meat quality, and sensory-panel outcomes.

For future validation, chicken type, production category, rearing period, dietary lipid source, meat portion, and fatty-acid composition should be defined first. Carcass traits, texture, oxidative stability, and volatile lipid-oxidation markers can then be assessed under standardized preparation conditions, followed by direct quantification of candidate nonvolatile markers such as 4HNE and 4HHE in free, lipid-associated, and protein- or peptide-bound forms. Receptor-oriented assays should be reserved for the final stage, after chemical data have been linked with instrumental meat-quality traits and sensory-panel outcomes.

### 8.5. Optional Receptor-Oriented Validation

Receptor assays come later, if the chemical and sensory data justify them. Transient receptor potential ankyrin 1 is an electrophile-sensitive somatosensory channel, and 4HNE may be considered a candidate electrophilic ligand for future validation [45]. This possibility justifies possible follow-up assays, but it does not show that cooked poultry samples contain receptor-relevant 4HNE concentrations or produce measurable oral sensory effects.

Sensory descriptors such as oral lingering, mouthfeel persistence, thickness, or continuity may be useful in future panel work, but they should not be equated with a confirmed receptor mechanism. Thus, 4HNE and 4HHE should first be measured as chemical markers in cooked meat, whereas TRPA1- or CaSR-related assays only become relevant after production context, cooked-matrix chemistry, and sensory endpoints have been connected.

## 9. Sensory Evaluation as a Meat-Quality Endpoint in Local Chicken Production Systems

Sensory evaluation is the stage at which production traits, instrumental meat-quality measurements, and meat composition are tested as consumer-relevant outcomes. In local chicken studies, it functions as a formal meat-quality endpoint rather than as an impression of regional cuisine. Chicken meat is heterogeneous, and sensory results depend strongly on test design, panel training, sample preparation, cooking procedure, cutting, presentation, serving temperature, and ethical protocol [46,47,48,49]. The descriptors therefore need to be defined in advance and linked, where possible, to instrumental and chemical measurements. Core endpoints include firmness, juiciness, aroma intensity, taste intensity, aftertaste, and overall palatability, with additional texture- or aroma-related terms included only when they can be evaluated reproducibly.

Trained descriptive panels, preference testing, and consumer-acceptance studies address different sensory questions and should not be interpreted interchangeably. In this review, descriptive sensory evaluation reported in previous studies is used only as empirical evidence for meat-quality descriptors, whereas preference and consumer-acceptance data are treated as separate outcomes that require dedicated experimental designs.

Comparisons of sensory findings among studies require caution because panel size, replication, blinding, sample presentation, and statistical design can substantially influence sensory outcomes. Therefore, sensory results cited in this review are used only as contextual descriptors rather than as directly comparable quantitative evidence across studies.

Sensory evaluation methodologies are broadly categorized according to the International Organization for Standardization (ISO) into three major groups: descriptive analysis [46], affective/hedonic tests [47], and discrimination tests [48,49]. These ISO standards provide internationally accepted definitions and procedural guidance for profiling sensory attributes, assessing consumer acceptance, and determining perceptible differences between products. In food science and in the quality-assurance and research-and-development activities of food companies, descriptive analysis is especially useful because it provides a structured assessment of taste, aroma, and texture. Discrimination-based sensory methods can also support product differentiation when their protocols and limitations are clearly described [48,49].

In addition, heterogeneity in intrinsic muscle properties—such as fiber structure, connective-tissue content, and muscle maturity—can influence sensory interpretation. Trained panelists may perceive firmness differences early during tasting, and these differences can affect the interpretation of subsequent taste and aroma descriptors. Therefore, texture should be measured instrumentally and controlled during sensory preparation whenever possible [18,22,23,24]. To minimize texture-related bias when the objective is taste or aroma comparison, mincing the meat is a practical approach [16,17], as it reduces structural differences and allows for more focused evaluation of taste and aroma.

Cross-study comparisons in this review are limited by heterogeneity in breed background, production system, slaughter age, diet, analytical workflow, storage condition, cooking model, and sensory protocol. These factors can influence fatty-acid composition, lipid-oxidation products, instrumental meat-quality traits, and sensory outcomes, and therefore reported differences should be interpreted as context-dependent rather than directly comparable across studies. Sensory results are further affected by panel type, panel size, replication, blinding, sample presentation, and statistical design, so they are used here only as contextual descriptors unless supported by matched experimental conditions.

Controlled dietary studies provide the clearest experimental evidence linking dietary lipid source, meat fatty-acid composition, and sensory response. Kiyohara et al. reported that arachidonic-acid-enriched oil improved the taste of Hinai-jidori thigh meat, and Takahashi et al. later observed a comparable effect in broiler thigh meat [16,17]. In both cases, dietary manipulation was connected to thigh-meat fatty-acid composition and sensory-panel outcomes. The reported changes in taste intensity, aftertaste, and overall palatability suggest that diet-induced changes in meat lipid composition can contribute to integrated sensory quality. The evidence remains portion-specific, however, and does not establish the same effect in breast meat or identify a receptor mechanism.

Other JAS-certified chickens offer supporting, but still limited, evidence. Aizu-jidori breast meat has been reported to contain a higher proportion of arachidonic acid than broiler breast meat, and Choshu-Kurokashiwa thigh meat was enriched in arachidonic acid compared with commercial broilers and was distinguished from them by trained sensory analysis [12,13]. These findings show that fatty-acid composition and sensory differentiation are not unique to Hinai-jidori. They also reinforce the need to evaluate chicken type, production context, meat portion, fatty-acid profile, texture, and sensory-panel results together rather than treating any one variable as a stand-alone explanation.

Future sensory work should be designed around a clearly defined production context and matched sample preparation. Meat portions and standardized cooked edible fractions should be compared under controlled cooking, serving-temperature, and presentation conditions. Firmness, juiciness, aroma intensity, taste intensity, aftertaste, and overall palatability should be prioritized, while temporal descriptors such as persistence or continuity should be used only when the panel protocol supports them. Chemical or receptor-oriented hypotheses should enter the interpretation only after sensory differences have been reproducibly linked to production context, fatty-acid composition, oxidative-stability markers, and instrumental meat-quality traits.

## 10. Future Poultry Experiments for Production-Based Meat-Quality Validation

Future experiments should begin with defined production categories and matched comparisons among JAS-certified chickens, other Asian local chickens, and commercial broilers. Key variables include chicken type, genetic line, dietary lipid source, slaughter age, rearing system, meat portion, storage, and standardized sample preparation. Breast and thigh meat should be analyzed separately, with fatty-acid composition, oxidative stability, instrumental meat-quality traits, volatile and nonvolatile markers, and sensory-panel outcomes measured under the same experimental design.

Table 4 translates this approach into an experimental-design outline by linking production context with meat-quality measurements, sensory endpoints, and downstream validation options.

Future studies should adopt factorial or otherwise controlled experimental designs that separate genotype, slaughter age, diet, and rearing-system effects. These designs should combine fatty-acid profiling, volatile analysis, standardized heating conditions, and controlled sensory evaluation under matched sampling and analytical workflows.

Within this design, the AA/LA → 4HNE → TRPA1 sequence should be treated only as a downstream validation route, not as the conclusion itself. AA/LA should first be tested as a meat lipid trait interpreted within a production context, followed by direct 4HNE measurement and receptor-oriented assays only when chemical and sensory evidence justify them.

In practice, this design tests whether differences in meat lipid traits are reflected in chemical markers, instrumental meat-quality traits, and sensory outcomes under matched portion and cooking conditions.

## 11. Conclusions

Asian local chicken meat quality should be interpreted as a production phenotype shaped by chicken type, native-breed ancestry, rearing period, husbandry conditions, dietary lipid source, meat portion, growth, and muscle traits. JAS-certified chickens provide a regulated production context for comparison with commercial broilers, while AA/LA provides a measurable lipid trait for evaluating differences in meat fatty-acid composition. These lipid traits should be interpreted together with portion-specific texture, oxidative stability, instrumental meat-quality traits, and sensory-panel outcomes.

In this review, established biochemical relationships among fatty-acid composition, oxidation pathways, and aldehyde formation are distinguished from receptor-based sensory hypotheses, which remain exploratory and require targeted validation. Cooking-derived volatile aldehydes, 4HNE, 4HHE, and TRPA1- or CaSR-related assays do not yet explain local chicken palatability by themselves. They become useful only when they are tied to measured meat-quality differences, sensory data, and retained chemical markers in cooked edible fractions. Future studies should therefore first establish meat-quality and sensory differences under a clearly defined production context and matched conditions, and then test whether retained oxidation products and receptor assays add mechanistic support.

## Figures and Tables

**Table 1:** Selected AA and LA proportions and AA/LA values in skinless breast and thigh meat of broiler and JAS-certified chickens compiled from the Local Chicken Characteristics Elucidation Report and Report II.

Chicken Type	Breast AA (%)	Breast LA (%)	Breast AA/LA	Thigh AA (%)	Thigh LA (%)	Thigh AA/LA
Broiler	3.3	13.6	0.24	1.6	15.2	0.11
Tokyo Shamo	7.8	13.1	0.60	4.9	17.2	0.28
Nagoya Cochin	8.2	13.8	0.59	3.0	19.2	0.16
Hinai-jidori	7.8	12.0	0.65	2.8	15.7	0.18
Hakata Jidori	7.5	12.0	0.63	3.1	13.0	0.24
Aizu-jidori	8.1	12.0	0.68	3.0	13.5	0.22
Amakusa Daioh cross	13.1	14.7	0.89	4.3	18.6	0.23
Miyazaki Jitokko	6.8	16.9	0.40	3.3	20.3	0.16

Note: Values are percentages of total fatty acids in skinless meat. AA and LA denote arachidonic acid and linoleic acid, respectively. AA/LA values were calculated from the corresponding AA and LA proportions in each meat portion. Except for broiler, all chicken entries are treated here as selected JAS-certified chicken data compiled from available reports, not as a complete representation of all JAS-certified chickens. Tokyo Shamo, Nagoya Cochin, Hinai-jidori, and Hakata Jidori data were taken from the Local Chicken Characteristics Elucidation Report [14]. Broiler, Aizu-jidori, Amakusa Daioh cross, and Miyazaki Jitokko data were taken from the Local Chicken Characteristics Elucidation Report II [15]. Both reports provide fatty-acid composition measured using identical analytical protocols (skinless breast and thigh, gas chromatography) under standardized production conditions. Values shown in the table are direct extractions from the original datasets without recalculation, allowing comparison of AA, LA, and AA/LA ratios across breeds under consistent measurement criteria.

**Table 4:** Production and experimental factors, meat-quality endpoints, sensory-panel endpoints, and validation approaches for Asian local chicken meat-quality studies.

Production or Experimental Factor	Biological or Meat-Quality Relevance	Measurable Meat-Quality Endpoints	Sensory-Panel Endpoints	Validation Approach
Production category	Regulated comparison framework	Carcass traits, meat composition, FA profile, texture	Palatability, product differentiation	Matched JAS/local/broiler comparisons
Chicken type and native-breed ancestry	Growth, muscle development, lipid metabolism	Body weight, muscle traits, collagen, IMF, FA profile	Firmness, chewiness, characteristic flavor	Chicken-type-, line-, or category-based comparisons
Rearing period and slaughter age	Muscle maturation, collagen, postmortem traits	Shear force, collagen, cooking loss, WHC, pH	Firmness, chewiness, juiciness	Age- and production-matched trials
Rearing system and husbandry conditions	Activity, management context, carcass traits	Carcass yield, muscle traits, texture, oxidative stability	Texture, juiciness, aroma, palatability	Controlled rearing-system comparisons
Dietary lipid source	Lipid metabolism and oxidative stability	AA, LA, AA/LA, PUFA profile, IMF, oxidation markers	Taste intensity, aftertaste, aroma, palatability	Controlled dietary comparisons with FA, meat-quality, and sensory data
Meat portion	Muscle structure, lipid pool, skin/fat contribution	Breast/thigh FA profile, IMF, collagen, volatile/nonvolatile markers	Firmness, juiciness, aroma, taste, aftertaste	Portion-specific analysis
Storage and standardized cooking model	Oxidation, volatile recovery, edible-fraction chemistry	Cooking loss, texture, volatile aldehydes, 4HNE/4HHE	Aroma, taste, aftertaste, temporal descriptors	Standardized storage, heating, preparation, and serving
Sensory evaluation design	Link to consumer-relevant outcomes	Integrated instrumental, FA, volatile, nonvolatile, and texture data	Firmness, juiciness, aroma, taste, aftertaste, palatability	Trained panels with controlled serving and predefined descriptors
Optional downstream receptor validation	Final mechanistic testing	Receptor-relevant marker concentrations	Validated persistence or somatosensory descriptors	Optional TRPA1/CaSR testing where justified

Note: The table is intended as a conceptual experimental-design outline rather than a list of confirmed causal relationships. Abbreviations: JAS, Japanese Agricultural Standard; FA, fatty acid; IMF, intramuscular fat; WHC, water-holding capacity; AA, arachidonic acid; LA, linoleic acid; AA/LA, arachidonic-acid-to-linoleic-acid ratio; PUFA, polyunsaturated fatty acid(s); 4HNE, 4-hydroxy-2-nonenal; 4HHE, 4-hydroxy-2-hexenal; TRPA1, transient receptor potential ankyrin 1; and CaSR, calcium-sensing receptor.

## Data Availability

No new data were created or analyzed in this study. Data sharing is not applicable to this article.

## Abbreviations

The following abbreviations are used in this manuscript:

AA	arachidonic acid
AA/LA	arachidonic-acid-to-linoleic-acid ratio
4HHE	4-hydroxy-2-hexenal
4HNE	4-hydroxy-2-nonenal
CaSR	calcium-sensing receptor
FA	fatty acid
GC–IMS	gas chromatography–ion mobility spectrometry
IMF	intramuscular fat
IMP	inosine 5′-monophosphate
JAS	Japanese Agricultural Standard
LA	linoleic acid
PUFA	polyunsaturated fatty acid(s)
TRPA1	transient receptor potential ankyrin 1
WHC	water-holding capacity
